# Making sense of GWAS: using epigenomics and genome engineering to understand the functional relevance of SNPs in non-coding regions of the human genome

**DOI:** 10.1186/s13072-015-0050-4

**Published:** 2015-12-30

**Authors:** Yu Gyoung Tak, Peggy J. Farnham

**Affiliations:** Department of Biochemistry and Molecular Biology, Norris Comprehensive Cancer Center, Keck School of Medicine, University of Southern California, Los Angeles, CA 90089 USA

**Keywords:** GWAS, Enhancers, Non-coding SNPs, Genome engineering

## Abstract

Considerable progress towards an understanding of complex diseases has been made in recent years due to the development of high-throughput genotyping technologies. Using microarrays that contain millions of single-nucleotide polymorphisms (SNPs), Genome Wide Association Studies (GWASs) have identified SNPs that are associated with many complex diseases or traits. For example, as of February 2015, 2111 association studies have identified 15,396 SNPs for various diseases and traits, with the number of identified SNP-disease/trait associations increasing rapidly in recent years. However, it has been difficult for researchers to understand disease risk from GWAS results. This is because most GWAS-identified SNPs are located in non-coding regions of the genome. It is important to consider that the GWAS-identified SNPs serve only as representatives for all SNPs in the same haplotype block, and it is equally likely that other SNPs in high linkage disequilibrium (LD) with the array-identified SNPs are causal for the disease. Because it was hoped that disease-associated coding variants would be identified if the true casual SNPs were known, investigators have expanded their analyses using LD calculation and fine-mapping. However, such analyses also identified risk-associated SNPs located in non-coding regions. Thus, the GWAS field has been left with the conundrum as to how a single-nucleotide change in a non-coding region could confer increased risk for a specific disease. One possible answer to this puzzle is that the variant SNPs cause changes in gene expression levels rather than causing changes in protein function. This review provides a description of (1) advances in genomic and epigenomic approaches that incorporate functional annotation of regulatory elements to prioritize the disease risk-associated SNPs that are located in non-coding regions of the genome for follow-up studies, (2) various computational tools that aid in identifying gene expression changes caused by the non-coding disease-associated SNPs, and (3) experimental approaches to identify target genes of, and study the biological phenotypes conferred by, non-coding disease-associated SNPs.

## Introduction: the GWAS conundrum

Considerable progress towards an understanding of complex diseases has been made in recent years due to the development of high-throughput genotyping technologies. Using microarrays that contain millions of single-nucleotide polymorphisms (SNPs), Genome Wide Association Studies (GWASs) have identified SNPs that are associated with many complex diseases or traits [1]. Such studies rely on differences in the frequency of a specific SNP in, for example, healthy (or control) vs. diseased (or case) populations. To date, ~84.7 million validated SNPs have been identified in human populations [2]. GWAS arrays do not contain all mapped SNPs; rather they contain only index SNPs that represent SNPs in the same linkage disequilibrium (LD) block. However, it is estimated that they do capture most human genome variation through haplotype-based SNP imputation [3, 4]. The SNPs identified by GWAS that are statistically significantly over-represented in the disease (or case) populations are called risk-associated SNPs and genomic regions containing the SNPs are called risk loci for that particular disease. As of February 2015, 2111 different association studies have identified 15,396 index SNPs associated with various diseases and traits (http://www.genome.gov/gwastudies), with the number of identified SNP-disease/trait associations increasing rapidly in recent years [1]. However, it has been difficult for researchers to understand disease risk from GWAS results.

**Fig. 1:**
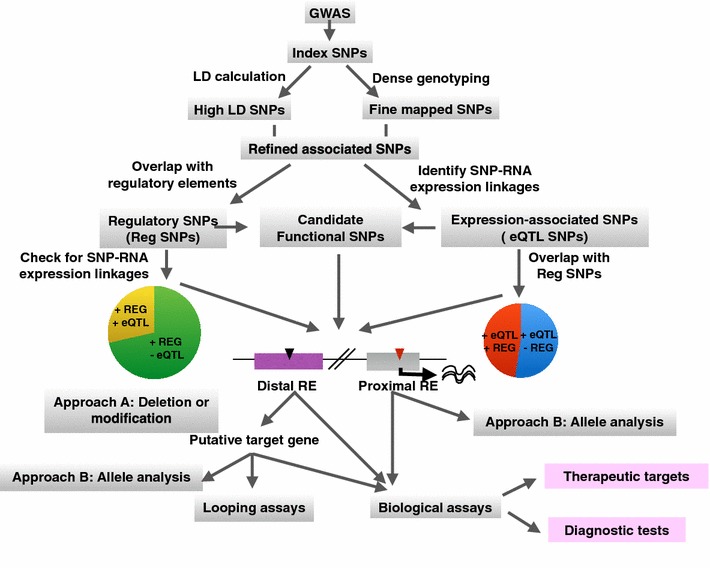
Making sense of GWAS: an overview. Shown is a flow chart of analytical and experimental steps that can be followed to understand how a non-coding SNP can be associated with an increased risk for a specific disease. Index SNPs are identified using GWAS arrays and then expanded to a larger set of SNPs (termed Refined Associated SNPs) using LD scores and fine-mapping. These Refined Associated SNPs are then prioritized using functional annotation to identify Regulatory SNPs (Reg SNPs) or linkage to allele-specific gene expression to identify eQTL SNPs, producing a set of Candidate Functional SNPs. The Candidate Functional SNPs can either be studied directly or further refined by testing the Regulatory SNPs for possible SNP-RNA linkages or by testing the eQTL SNPs for functional annotation. If a Candidate Functional SNP (*yellow arrowhead*) lies within a distal regulatory element, it can be deleted or modified using genomic nucleases or epigenomic toggle switches (*Approach A*); putative target genes are then identified using RNA-seq. Distal regulatory elements that cause changes in gene expression when deleted or modified can then be studied using allele-specific analyses (*Approach B*); promoters harboring risk-associated SNPs (*pink arrowhead*) can be directly studied using Approach B. As described in the text, cells deleted for the distal regulatory elements can be used to identify an appropriate phenotypic assay for analysis of the candidate target genes. Then, the genes that show expression changes that are linked to distal SNPs and the genes regulated by the promoter SNPs can be studied using those biological assays to identify possible therapeutic targets and/or candidates for diagnostic tests. Finally, looping assays can be performed to distinguish direct from indirect targets of the distal regulatory elements. It is important to note that a gene whose expression is indirectly affected by a non-coding SNP could be a more important diagnostic or therapeutic target than the directly affected gene

First, unlike a disease such as cystic fibrosis that is caused by mutations in the coding region of a gene, GWAS-identified disease-associated nucleotide differences are rarely found in coding regions. Instead, most disease-associated index SNPs are located in non-coding regions of the genome, equally proportioned between the intergenic and intronic compartments [5, 6]. However, it is important to consider that the GWAS-identified index SNPs actually serve only as representatives for all the SNPs in the same haplotype block, and it is possible that other SNPs in high LD with the GWAS-identified index SNPs are causal for the disease. Because it was hoped that disease-associated coding variants would be identified if the true casual SNPs were known, investigators began expanding their analyses to include more than just the index SNPs. A commonly used approach to investigate SNPs other than the index SNPs present on the standard GWAS array has been to use LD calculation [7–9] together with the 1000 Genomes Project reference panels from different populations [2, 10]. Such approaches have generally expanded the list of putative causal SNPs from less than 100 index SNPs for a particular disease or trait to several hundred associated SNPs (Fig. 1; High LD SNPs). For example, 727 SNPs are in high LD (*r*^2^ > 0.5) with 77 index SNPs linked to prostate cancer [11]. However, most of these LD-associated SNPs are also in non-coding regions of the genome. Similarly, SNPs correlated with 25 colon cancer risk-associated index SNPs were analyzed (using an *r*^2^ > 0.5); 13 correlated SNPs were located in exons (only 2 of which were predicted to be damaging to the protein structure), whereas 503 correlated SNPs were located in non-coding regions corresponding to promoters or enhancers [12]. Another approach called fine-mapping is also being used in attempts to move from the index SNP (which basically identifies a large genomic region) to a more refined list of putative causal SNPs located within the identified region. Fine-mapping studies employ dense genotyping arrays that contain all common SNPs within the previously identified risk loci, which together with imputation [7–9] allows investigators to perform a more complete analysis of the risk regions (Fig. 1; fine-mapped SNPs) [13]. However, genotyping at this fine scale requires large sample sizes to provide the statistical power needed to differentiate the candidate causal SNPs from the non-causal SNPs. In addition, creation of loci-specific genotyping arrays is quite expensive. Therefore, most fine-mapping analyses have been done by international consortia with shared interests for specific diseases or traits; examples include the Immunochip [14], the Metabochip [15], the iCOGs array [16], and the Oncoarray (http://epi.grants.cancer/gov/oncoarray/). The majority of fine-mapping studies have been performed using European-ancestry populations in which LD blocks are longer than in other populations and therefore there are many correlated SNPs per loci, exacerbating the problems related to a need for large sample sizes to separate true candidate causal SNPs from less significantly risk-associated SNPs [17, 18]. However, recent fine-mapping studies of trans-ethnic populations have shown better results in discovering candidate causal SNPs [4, 19–21]; trans-ethnic fine-mapping increases statistical power by increasing the number of samples and also helps avoid false positives due to confounding factors of population stratification. However, a recent multi-ethnic analysis of prostate cancer risk SNPs found that even after fine-mapping, most risk-associated SNPs are located in non-coding regions [21]. Thus, the GWAS field has been left with the conundrum as to how a single-nucleotide change in a non-coding region could confer increased risk for a specific disease. One possible answer to this puzzle is that the variant SNPs cause changes in gene expression levels rather than causing changes in protein function. This review provides a description of (1) advances in genomic and epigenomic approaches that incorporate functional annotation of regulatory elements to prioritize the disease risk-associated SNPs that are located in non-coding regions of the genome for follow-up studies, (2) various computational tools that aid in identifying gene expression changes caused by the non-coding disease-associated SNPs, and (3) experimental approaches to identify target genes of, and study the biological phenotypes conferred by, non-coding disease-associated SNPs.

## Making sense of GWAS

### Prioritization of SNPs associated with a specific disease using functional annotation

As noted above, not only do the vast majority (~93 %) of index SNPs in the GWAS catalog that have been associated with specific diseases or traits lie within non-coding regions, but also most SNPs in high LD with the risk-associated index SNPs and most SNPs identified by fine-mapping (Fig. 1; collectively identified as refined associated SNPs) are also located in non-coding regions. The current hypothesis is that one or more of these risk-associated non-coding SNPs cause changes in gene expression of a critical gene. However, functional follow-up experiments (described below) are both expensive and time-consuming and one cannot test each possible candidate SNP for causality. It is also important to note that although fine-mapping usually results in a smaller number of associated SNPs than does LD calculation, fine-mapping has only been performed for a relatively small number of disease-associated loci and therefore most investigators are left with the problem of a fairly large list of possible causal SNPs. Clearly, it is necessary to prioritize the list of refined associated SNPs for follow-up analyses.

One way to prioritize the list of SNPs is to identify those located in regulatory regions of the genome (Fig. 1; Regulatory SNPs). The first step in identifying Regulatory SNPs is to select from the list of Refined Associated SNPs those that lie within regulatory regions. There are several types of elements involved in transcriptional regulation including promoters, enhancers, and nuclear structure-associated elements such as CCCTC-binding factor (CTCF) binding regions; each of these elements has been associated with non-coding SNPs. A promoter is a user-defined region, usually described as several Kb surrounding a transcription start site (TSS) of a known coding or non-coding gene (active promoters can also be defined by certain histone marks or RNA analysis, if the datasets are available for that particular cell type). Thus, investigators can bioinformatically identify promoter SNPs. It is more difficult to identify SNPs within enhancers because, unlike promoters, they do not occur at a defined distance from a TSS. However, they can be identified by specific epigenomic profiles. Within recent years, consortia such as the Encyclopedia of DNA Elements (ENCODE) [22] and the Roadmap Epigenomics Mapping Consortium (REMC) [23, 24] have used a variety of genome-wide methods to study the chromatin state of non-coding regions in the human genome in hundreds of different cell types (e.g., primary cell lines, immortalized cell lines, and tissues). In these studies, enhancers have been identified using methods that detect open chromatin, specific histone modifications, and enhancer RNAs (eRNAs). For example, DNase-seq [25] has been used to identify DNase-hypersensitive regions (DHSs) that correspond to areas of open, accessible chromatin that contain binding motifs for transcription factors (TFs). Although DHSs are generally a few Kb in length, DNase footprinting (which combines deeply sequenced DNase-seq data with motif information) can help more precisely identify the critical nucleotides for TF occupancy within a DHS site [26, 27]. More recently, ATAC-seq, a method that employs an engineered Tn5 transposase to measure chromatin accessibility, has been used to define genomic maps of open chromatin; advantages of ATAC-seq include the requirement for fewer cells (500–50,000 cells) and fewer experimental steps, as compared to DNase-seq [28]. The entire set of DHSs includes promoter regions, distal enhancer regions, and sites of binding of structural TFs. To further refine the set of distal DHSs to include only active enhancers, investigators use the method of ChIP-seq and antibodies specific to histone modifications. For example, potentially active enhancers are identified as regions of open chromatin with flanking nucleosomes having histone 3 marked by monomethylation of lysine 4 (H3K4me1), whereas nucleosomes flanking fully active enhancers are marked by H3K4me1 and also by acetylation of lysine 27 on histone H3 (H3K27Ac) [22]; enhancers also sometimes have low levels of histone H3 trimethylated on lysine 4 (H3K4me3), a mark that is quite strong at promoters. The H3K27Ac mark at enhancers is likely a consequence of the binding of site-specific TFs (e.g., TCF7L2) that recruit histone acetyltransferases (HATs) such as EP300 and CBP. It is thought that the acetylation of the histones flanking a DHS increases the net affinity of other TFs to the region of open chromatin [29]. Thus, it seems logical that identifying active enhancers using TF ChIP-seq data would also be possible. However, considering the fact that ChIP-seq patterns have been identified for less than 150 TFs out of 1800 known TFs (and in only a few cell types), the combination of DHS and histone modifications is more commonly used to identify enhancers [25]. However, we note that recent studies have identified active enhancers using changes in levels of DNA methylation at regions distal from promoters [24, 30–34]. Finally, a different approach to identifying active enhancers has been used by the FANTOM5 project, which employed cap analysis of gene expression (CAGE) to discover active enhancers that produce bidirectional capped RNA. Notably, although very few enhancers were identified by this method, a high percentage of these enhancers were validated by reporter assays [35]. It is also important to note that enhancers are very cell-type specific and therefore enhancer mapping must be performed in the cell type(s) that are relevant to the disease under study.

Several studies have shown that index SNPs and/or correlated SNPs that are in high LD to the index SNPs are enriched in enhancer regions. For example, one study found that non-coding index SNPs from 426 GWASs are enriched in enhancers present in the relevant cell types and that several of the index SNPs created or disrupted TF motifs in the identified enhancers [36]. Also, Schaub et al. studied 4724 GWAS index SNPs associated with 470 different phenotypes using ENCODE data, showing that 36 % of the SNPs are in DHSs and 20 % are in a ChIP-seq peak in at least one cell line. When they extended their analyses to SNPs that are in high LD (*r*^2^ > 0.8) with the index SNPs, the overlap increased by over two-fold [26]. These findings are consistent with a recent study in which investigators used H3K27Ac ChIP-seq data from normal and colon cancer cells and found that 270 SNPs that have a high LD (*r*^2^ > 0.5) with 25 colorectal cancer index SNPs are located in H3K27Ac sites; when the SNPs were limited to distal regions they identified 68 unique enhancers [12]. Similarly, combining H3K27Ac and H3K4me1 ChIP-seq data and DNase-seq data from prostate cancer cells, Hazelett et al. identified 727 SNPs that were in high LD (*r*^2^ > 0.5) with 77 prostate cancer risk SNPs; of these, 663 SNPs were in putative enhancer regions [11]. Also, a recent fine-mapping study of Type 1 Diabetes (T1D) found that fine-mapped T1D-associated SNPs are localized in active enhancers of thymus, T and B cells, and CD34+ stem cells [37, 38].

The working model for establishment and maintenance of active enhancers is that TFs bind to the DNA, position the nucleosomes, and then serve to keep the region between the nucleosomes in an open conformation [29]. Thus, it is logical to assume that risk-associated regulatory SNPs would have a higher likelihood of causality if they disrupt or create a motif for a site-specific TF in the nucleosome-free region of an enhancer or DHS. Unfortunately, although progress has been made in identifying in vivo motifs for TFs using ChIP-seq data [39], the motifs for most site-specific TFs are not known. However, programs have been developed that allow investigators to incorporate information about the set of known TF motifs into SNP prioritization [40, 41]. Using such programs, Regulatory SNPs located in motifs of TFs known to be important in establishing or maintaining the phenotypic characteristics of specific cell types have been identified. For example, motifbreakR [41] can predict TF motif disruptions for a large number of provided SNPs using several different sources of TF motifs (see Table 1 for details). However, it should be noted that studies have shown that many risk-associated SNPs (index SNPs and SNPs that are in high LD to index SNPs) are not precisely located in the conserved binding motif of TFs but are in nearby regions [42, 43]. It is possible that such SNPs disrupt an as-of-yet unknown motif for a TF that has not yet been characterized by ChIP-seq. Additionally, another possibility is that sequences outside the core TF binding motif can affect TF binding. Recent work has suggested that the environment of the motifs, including sequence composition, DNA shape features, and an overall high similarity to the core- binding motif can contribute to binding affinity of TFs to various DNA sequences [44–47]. Finally, recent studies suggest that only a minority of the single-nucleotide changes in TF binding motifs that have been identified to date actually affect binding in vivo of a TF, with extensive context-dependent buffering of the possible effects on TF recruitment that could potentially occur due to changes in recognition motifs [48].

**Table 1:** Publicly available functional annotation programs

Tool	Type	Minimum input	Output	Epigenetic annotation file used	URL	PMID
HaploReg	Web server	rsID	Overlapping annotated features and TF motif disruption information for SNPs (input) and correlated SNPs with r^2^ >0.8	ChromHMM, DNase-seq, a library of position weight matrices (PWMs) from TRANSFAC, JASPAR, and protein binding array (PBM), and eQTL	http://www.broadinstitute.org/mammals/haploreg/haploreg_v3.php	22064851
RegulomeDB	Web server	rsID	Overlapping annotated features for SNPs (input) with scores which depend on the combination of overlapping annotated features and UCSC genome browser showing overlapping features	TF binding, Dnase-seq, FAIRE, DNase footprinting, eQTL, dsQTL, ChIP-exo and DNA methylation	http://www.regulomedb.org	22955989
FORGE	Web server	rsID	Overlapping DNase1 hotspots for SNP(input)	DNase1 hotspot	http://browser.1000genomes.org/Homo_sapiens/UserData/Forge	
rSNPBase	Web server	rsID or gene name	Proximal or distal transcriptional regulation, miRNA regulation, RNA binding protein mediated regulation, eQTL results for SNPs (input) and correlated SNPs (r^2^>0.8)	histone modification, TF bindings, CpG islands, RBP, miRNA data	http://rsnp.psych.ac.cn/	24285297
FunciSNP	R package	GWAS index SNP information (chrom:position, rsID, population)in tab-delimited file, biofeature information in .bed format, user‐defined *r* ^2^ value	Overlapping annotated features for index SNP(input) and correlated SNPs which r^2^ values are user‐defined	Any biofeature annotation information in .bed format	https://github.com/labrazil/Coetzee_Seq_Analysis/tree/master/FunciSNP	22684628
GREGOR	A package run using perl code	A file containing single column of index SNPs, biofeature information in .bed format, user‐defined *r* ^2^ value	Prioritized variants based on overlap with selected regulatory regions, enrichment analysis with P‐values showing how index SNPs or correlated SNPs are enriched in annotated feature compared to control SNPs	Any biofeature annotation information in .bed format	http://csg.sph.umich.edu/GREGOR/index.php/site/index	25886982
Enlight	Web server	rsID, *P* value	Plots showing LD and overlapping annotated features for SNP (input)	chromHMM, histone modification, DNA methylation, TF bindings, eQTL, Hi-C or customized BED file for biofeatures	http://enlight.usc.edu/index.html	25262152
GWAS3D	Web server	rsID, *P-* value	TF motif analysis and overlapping annotated features for SNPs (input)	5C, Hi-C, ChIA-PET, ChromeHMM, H3K27Ac, p300, CTCF, DHS (Option for selecting cell lines relevant to disease)	http://jjwanglab.org/gwas3d	23723249
motifbreakR	R package	SNP information in .bed or .vcf format	Comprehensive TF binding sites disruption at SNPs (input)	TF motif information from ScerTF, FlyFactorSurvey, hpDI, UniPROBE, JASPAR, ENCODE, Homer, Factorbook, HOCOMOCO	https://github.com/Simon-Coetzee/motifBreakR--	26272984

Perhaps the TF for which the most ChIP-seq experiments have been performed is CTCF [49–51]. ENCODE, as well as many individual laboratories, have mapped CTCF binding in a large number of human cell types. Such studies have revealed that CTCF binds to promoter and enhancer regions, but it can also bind to regions of the genome that lack the histone modifications that specify active promoters and enhancers. For example, in Panc1 cells, 15 % of CTCF peaks are in promoters, 14 % are in enhancers, and 71 % are in neither promoters nor enhancers (M. Gaddis and P. Farnham, unpublished data). Topologically associating domains (TADs), which demarcate large chromatin regions that interact via looping, are enriched for CTCF binding sites at their boundaries, suggesting a role for CTCF-mediated looping in the maintenance of TADs [52]. CTCF is also thought to contribute to the overall 3-dimensional structure of chromatin by forming a loop through which distal enhancers and promoters can be brought into close proximity, perhaps leading to transcriptional activation of the linked promoter [51]. CTCF has also been shown to serve as insulator that interferes with the interaction between an enhancer and a promoter and to block chromosome position effects of transgenes [51]. Thus, regulatory SNPs that disrupt or create a CTCF site may be of high priority for follow-up analyses. We note that a combined analysis of GWAS SNPs for numerous complex diseases and traits did not show an enrichment for CTCF sites [48]. However, it is possible that only a subset of the CTCF sites are functionally relevant in relation to GWAS variation (e.g., the structural CTCF sites may have a different enrichment score than the CTCF sites that fall within regulatory elements). It is also possible that variation at CTCF sites may play a role only in specific diseases. For example, a recent GWAS of a Chinese population identified 3 index SNPs statistically associated with increased risk of lung cancer that are located within CTCF ChIP-seq peaks in the A549 lung cancer cell line [53]. In addition, Ding et al. identified statistically significant allele-specific CTCF binding data from 50 lymphoblastoid cells lines [54] which were genotyped as a part of the 1000 Genomes Project, providing a source of prioritized SNPs to study the involvement of CTCF in disease risk [55]. Interestingly, only 25 % of these genetic variants are exactly in the CTCF motif; however, most are located within 1 Kb of the motif [55]. This finding is consistent with the studies described above showing that many risk-associated SNPs are not in the conserved binding motif of TFs but are in nearby regions. Of course, it is not yet known if the SNPs that are nearby, but not in, CTCF motifs are functionally relevant.

Finally, SNPs located within CpG sites have been studied for their relationship to disease. Clearly, if a CpG site within a known motif for a TF is identified as a disease-associated SNP, it could alter gene regulation simply by changing the affinity of the TF for that region. In fact, several TFs do harbor CpG dinucleotides at critical positions in their motifs [6, 56]. However, CpGs can also regulate gene expression in a more region-specific way. CpG island methylation of promoter regions of tumor-suppressor genes is one of the driving factors for cancer development [57]. In addition, recent studies have shown that hyper- and hypo-methylation of distal elements can be linked to tumor-specific changes in gene expression [30]. Increased methylation of a promoter or enhancer is generally thought to lead to transcriptional repression, whereas decreased methylation is thought to lead to gene activation. Thus, a single allele change at a SNP (which disrupts or increases binding of a TF by affecting DNA methylation) can lead to an altered epigenetic pattern of a larger region. Measuring methylation levels at 22,290 CpG dinucleotides in lymphoblastoid cell lines of 77 individuals from the HapMap project, Bell et al. found 180 CpG sites in 173 genes that are associated with SNPs located within 5 Kb [58]. Additionally, several diseases have been reported to be linked to aberrant SNP-associated methylation at CpGs in promoter regions [59–61]. For example, Hitchins et al. found that a single-nucleotide variant in the 5′ UTR of the *MLH1* gene resulted in increased methylation of the promoter, leading to transcriptional repression. It has been suggested that the variant SNP decreases recruitment of a TF, causing loss of protection from methylation on nearby CpG sites, thus leading to Lynch syndrome.

**Fig. 2:**
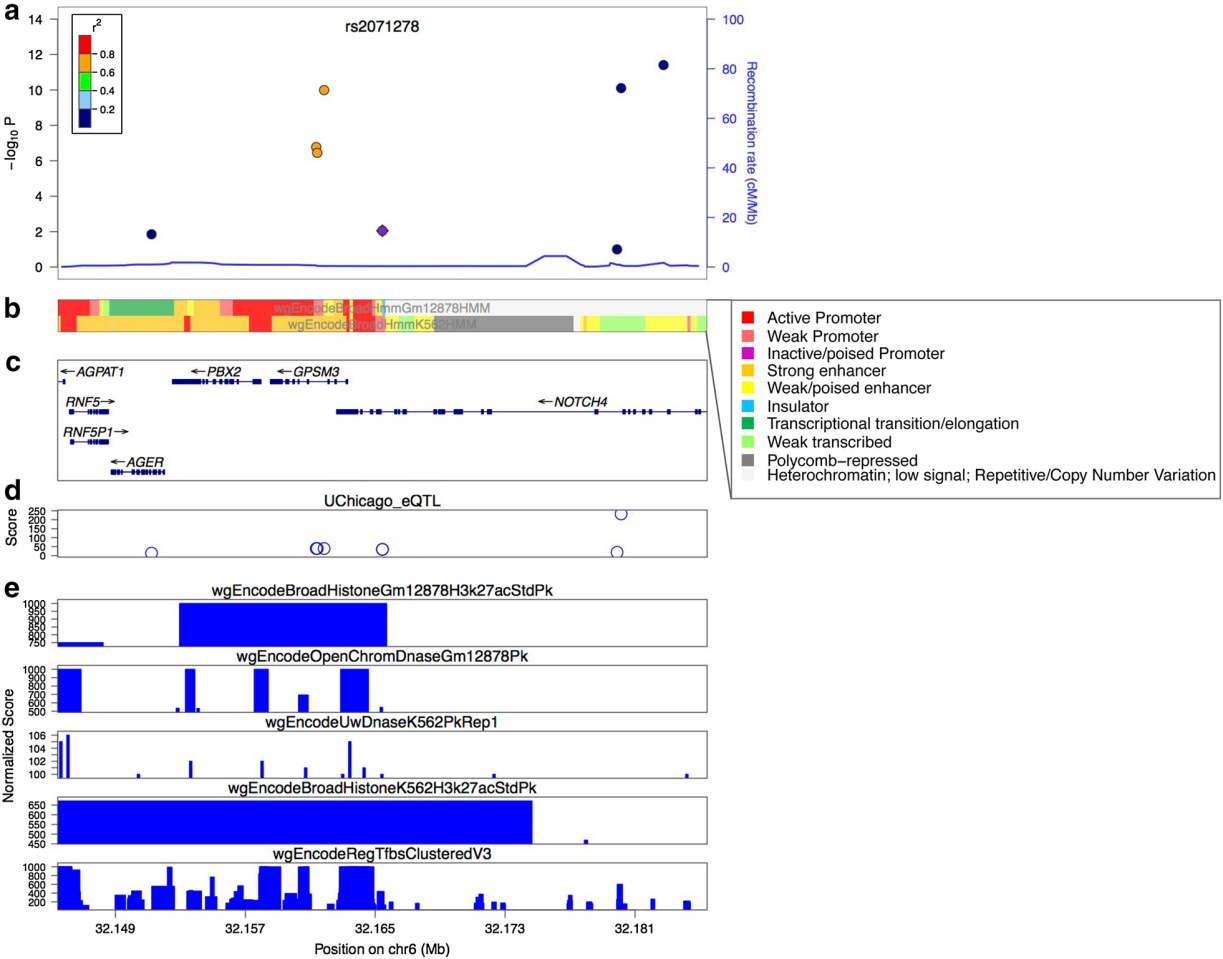
Prioritizing SNPs using functional annotation. Shown is a figure produced using the Enlight program. **a** Shown is an index SNP (rs2071278, indicated by the purple diamond) for Rheumatoid Arthritis and correlated SNPs within ±20 Kb; the high LD SNPs (*r* ^2^ > 0.8) are indicated in *orange*. **b** Shown is the chromHMM segmentation for the region, with the colors (defined in the *inset box*) indicating the different chromatin states for that region in the blood cell lines, GM12878 and K562; note that the High LD SNPs fall into enhancer categories (*yellow bars*). **c** Shown are the genes within the region. **d** Shown is an eQTL plot with scores based on −log_10_ *P* values, taken from the UChicago eQTL browser. **e** Shown is H3K27Ac and DNase-seq data for GM12878 and K562 and the TFs ChIP-seq track from the ENCODE browser

As described above, identification of Regulatory SNPs requires investigation as to whether any of the Refined Association SNPs fall within promoters, enhancers, TF binding sites, or CpG dinucleotides. Although one could determine if any of the relatively small set of index SNPs for a particular disease is located within a mapped regulatory element by simply visualizing the location of the SNP and the location of functional elements on a genome browser, it would be quite laborious to do this for the many hundreds of the SNPs in high LD with the index SNPs. Therefore, several different programs have been developed that integrate genetic information (genotyping and imputation data for GWAS index SNPs and SNPs in LD to index SNPs) with epigenetic information (generated by DNase-seq, ChIP-seq, or DNA methylation assays) and chromatin interaction data. Listed in Table 1 are some of the publicly available functional annotation programs; each program has its own advantages and disadvantages. For example, Regulome DB [62] and HaploReg [63] share similar features, automatically providing all possible epigenetic information for all available cell types and tissues for the input SNPs (the epigenetic maps are derived from the ENCODE and REMC databases). However, neither program has options for analyzing only the relevant cell types for the disease-associated SNPs. In contrast, FunciSNP [64], GREGOR [65], and Enlight [66] allow users to add their specific epigenetic data from the cell type of interest (which may not be in the public databases), providing a better prioritization of the regulatory SNPs. Of note, GWAS3D [67] and Enlight [66] include an automatic analysis of chromatin interaction features (although such data are not yet available for many cell types), and Enlight automatically generates plots showing LD information and overlapping annotated features (Fig. 2).

### Prioritization of SNPs associated with a specific disease by linking to gene expression

A second way to prioritize risk-associated SNPs is to focus on the subset that show allele-specific gene expression differences, as determined using population-based methods. The population-based methods identify expression quantitative trait loci (eQTL), which are defined as genomic regions that harbor one or more nucleotide variants that correlate with differences in gene expression [68]. We note that although eQTLs are said to identify “loci,” most investigators use this term to refer to specific nucleotides (i.e., SNPs) that correlate with differences in gene expression [68]. Expression-associated SNPs (Fig. 1; eQTL SNPs) can be statistically significantly associated with genes that are located in a genomic region near to or far from the SNP in question. If associated with a nearby gene, the relationship is termed a “local eQTL,” whereas SNPs associated with genes located farther away on the same chromosome or on different chromosomes are called “distal eQTLs.” In many cases, local eQTLs work as cis-eQTLs, which directly affect expression of nearby genes (usually limited to genes within 250 Kb to 1 Mb) in an allelic-specific manner [68, 69]. In contrast, trans-eQTLs cannot be applied to the study of allele-specific gene expression because they likely affect expression of the identified gene as a secondary consequence of changes in direct target genes. Most trans-eQTLs are distal eQTLs, being associated with genes found far from the SNP on the same chromosome or on different chromosomes [70]. However, it should be noted that some trans-eQTLs are local eQTLs, even though nearby the SNP under study, the associated gene is affected as a secondary consequence of gene expression changes of a direct target gene. Most studies focus on cis-eQTLs [70] because trans-eQTLs require multiple testing to gain statistical power [71].

For eQTL analyses, SNPs are mapped using a genotyping array and mRNA abundance is measured by microarray or, more commonly in recent studies, by RNA-seq using hundreds of samples from cell lines or tissues that are relevant to the disease or traits under study. Statistical methods are then used to associate SNPs with transcripts to identify eQTLs [69]; sources of eQTL databases are listed in Table 2. It is important to note that all mapped eQTL SNPs (especially those from large studies) are not linked to disease; in other words, some SNPs associated with gene expression were not identified via GWAS. However, many studies have revealed that eQTLs can be identified for some GWAS risk loci (testing index SNPs or SNPs in high LD with the index SNPs); in these cases, the association of the SNP and expression of nearby genes was identified in a trait or disease-specific manner [72–75]. For example, Type 2 Diabetes (T2D) index SNPs and high LD SNPs (*r*^2^ > 0.9) are enriched in the set of eQTL SNPs identified using liver and fat tissues [73]. Also, eQTL SNPs identified using gene expression datasets from blood showed enrichment for association with autoimmune disease, but not with bipolar disorder or T2D [76]. It is important to note that the sets of genes located nearby GWAS-identified SNPs are not always highly concordant with eQTL-associated genes [76], suggesting that some GWAS signals affect genes that are far away. Therefore, we cannot conclude that the target genes of GWAS SNPs are the same genes identified by cis-eQTL SNPs. For example, Musunuru et al. [77] used GWAS information to identify a risk SNP at 1p13 that is associated with both plasma low-density lipoprotein cholesterol (LDL-C) and myocardial infarction (MI). Also, they used eQTL analysis of liver gene expression datasets to determine if risk SNPs in the 1p13 region are associated with nearby genes, finding that two GWAS-identified risk-associated SNPs (rs646776 and rs12740374) were in eQTL with the *SORT1* gene. The authors suggest that the minor allele of rs12740374 creates a C/EBP binding site and results in increased *SORT1* expression, which contributes to the risk for LDL-C and MI. However, it should be noted that *SORT1* is not the nearest gene to rs12740374 and is located 123 Kb from the risk-associated SNP.

**Table 2:** Sources of eQTL databases

Tool	Features	URL	PMID
NCBI eQTL browser	cis‐eQTL from liver, lymphoblastoid, brain	http://www.ncbi.nlm.nih.gov/projects/gap/eqtl/index.cgi	
seeQTL	browser for cis‐eQTL, and trans-eQTL from lymphoblastoid, brain, monocyte	http://www.bios.unc.edu/research/genomic_software/seeQTL/	22171328
Chicago eQTL	QTL (eQTL, dsQTL, trQTL, exonQTL) from lymphoblastoid, brain, liver, fibroblast, T‐cells	http://eqtl.uchicago.edu/cgi--‐bin/gbrowse/eqtl/	
GTEx Portal	>60 tissues eQTL data and eQTL IGV browser	http://www.gtexportal.org/home/	25954001
GeneVar	>5 tissues eQTL, meQTL data and visualization	https://www.sanger.ac.uk/resources/software/genevar/	20702402
Blood eQTL	Blood cis- and trans-eQTLs	http://genenetwork.nl/bloodeqtlbrowser/	24013639
Geuvadis	QTL (eQTL,mirQTL, trQTL) from lymphoblastoid cell lines	http://www.ebi.ac.uk/Tools/geuvadis--‐das/	24037378

*mirQTL* miRNA QTL, *trQTL* transcript ratio QTL, *dsQTL* Dnase I sensitivity QTL

Although some eQTLs are shared across different cell types, most eQTL associations are cell-type specific [78, 79]. These cell-type specific eQTLs are often quite far from the gene they are associated with and tend to have small effects on gene expression, reflecting the characteristics of enhancer elements [78]. Using epigenetic information from ENCODE and REMC to functionally annotate 4085 intergenic eQTLs, investigators showed that the eQTLs which have the highest significance per gene are enriched in TF binding sites, enhancers, promoters, and open chromatin. A recent study identified enrichment of eQTL SNPs in distal elements, but the SNP-gene expression linkage only appeared upon immune stimulation of naïve monocytes [80], suggesting that new enhancers harboring eQTL SNPs were created by immune stimuli. Several studies have suggested that changes in TF binding are a major result of cell-type specific eQTLs, leading to changes in chromatin structure, histone modification, or methylation, with resultant changes in gene expression [81]. In a recent study correlating RNA-seq data from 103 matched tumor and normal colon mucosa samples from Danish patients with germline genotyping from 90 patients, investigators found that many of the identified eQTLs are tumor specific. Using ChIP-seq data from a colon cancer cell line, they concluded that the tumor-specific eQTLs are associated with binding of several TFs that show increased expression in tumors [82]. Other evidence supporting an important role for TF binding in the mechanism by which eQTLs function is provided by meQTLs, defined as CpG sites in which DNA methylation changes have association with SNPs that are several Kb away [83]. A recent study showed that 23 SNPs out of 109 cancer GWAS SNPs from 13 different cancer types had associations with methylation status [84]. Banovich et al. showed that meQTLs are frequently associated with changes in histone modification, DNase hypersensitivity, chromatin accessibility, and expression changes in nearby genes. As described above, meQTLs are thought to affect TF binding, which in turn influences DNA methylations at nearby CpG sites [83]. In the cases where meQTLs are eQTLs, a positive correlation between methylation and expression was shown when meQTLs are not near a TSS (median distance of ~7 Kb) and a negative correlation between methylation and expression was shown when meQTLs are near a TSS (median distance of ~1 Kb), which is consistent with findings that active promoters show low DNA methylation whereas bodies of actively transcribed genes show high DNA methylation [83, 85].

### Experimental approaches to identify target genes of regulatory SNPs and eQTL SNPs

Although investigators often use either functional annotation or eQTL to identify prioritized SNPs (Fig. 1; collectively referred to as Candidate Functional SNPs), using a combination approach may help rank the individual lists for follow-up study. The set of Regulatory SNPs (especially those obtained using high LD and not fine-mapping) is usually larger than the set of eQTL SNPs. Therefore, determining if any of the large set of enhancers that harbor risk-associated SNPs are also in eQTL with one or more genes may identify a subset of risk-associated enhancers that have a higher probability of having an impact on gene expression. Similarly, although the set of eQTL SNPs is usually not large, it is difficult to perform functional follow-up studies of the entire set. Therefore, determining which of the eQTL SNPs are also located in a regulatory region could help prioritize the list. Having identified a set of Regulatory and/or eQTL SNPs, the next logical step would seem to be functional follow-up studies of the genes regulated by the SNP-harboring elements. However, it is not easy to determine the actual target gene of a regulatory element. It is a commonly held assumption that a risk-associated SNP that falls within a promoter region influences expression of that particular gene. In fact, if the gene in question has a known biological function consistent with the possibility that it may influence cellular phenotype in a manner consistent with the disease being studied, then investigators often go straight to studying that gene. However, some have postulated that promoters can interact with other promoters [86] and can also have enhancer activity, influencing the expression of other genes [87]. Thus, it may be premature to assume that SNPs located near to the 5′ end of a gene only influence the regulation of that particular gene. It is even more difficult to predict what gene is directly regulated by an enhancer because they are located distal from a TSS, can regulate genes in an orientation-independent manner, and, most importantly, can skip over nearby genes to regulate genes farther away. Thus, although one hypothesis is that the gene nearest to a promoter or an enhancer that harbors a regulatory SNP is the disease-related gene, in most cases this hypothesis has not been proven (or even tested). However, there are unbiased approaches that can be used to experimentally identify target genes of the regulatory elements harboring Candidate Functional SNPs. This can be accomplished by manipulating the genomic region containing the SNPs in question and determining if expression of the putative target gene is in fact altered and/or by testing physical interactions between the region harboring the SNP and a putative target gene using looping assays.

*Deletion or epigenetic modification of distal regulatory elements harboring Candidate Functional SNPs* One approach towards identifying a target gene of a distal element is to delete or epigenetically modify the element and study subsequent effects on the transcriptome (Fig. 1; Approach A). We note that because deleting or inactivating an entire promoter region would automatically eliminate expression from that gene (making it difficult to determine the exact role of the SNP), analysis of Candidate Functional SNPs located in promoters should begin with specific targeting methods described below (Fig. 1; Approach B). A traditional method to study a distal regulatory element in the genome of cultured cells or in a mouse model has been to remove or replace a wildtype regulatory element with a mutated version using loxP and the Cre recombinase. In a recent study, the loxP-Cre recombination method was used to delete an enhancer from the mouse genome that is located within a region that corresponds to a region of the human genome harboring a colon cancer GWAS SNP [88]. Mice lacking this enhancer element were resistant to intestinal tumor formation, possibly due to down regulation of Myc, which is located 335 kb from the deleted sequence. Although these results are promising, there are disadvantages in using the loxP-Cre system. For example, cloning of the plasmids needed for homologous recombination is laborious and the insertion of the foreign loxP DNA sequence into the genome could potentially affect gene expression [89]. Fortunately, recently developed technologies that are based on zinc finger proteins (ZFPs), transcription activator-like effectors (TALEs), or the clustered regularly interspaced short palindromic repeats (CRISPRs) have allowed researchers to investigate functionality of genomic elements in the endogenous context in almost any organism [90]. Using these genomic engineering platforms, regulatory elements can be deleted from the genome without the introduction of exogenous sequences. In addition, the same genomic platforms can be used to epigenetically alter the genomic sequences containing a risk-associated SNP.

Regulatory elements harboring Candidate Functional SNPs can be deleted using zinc finger-based nucleases (ZFNs), TALE-based nucleases (TALENs), or CRISPR-associated protein 9 (Cas9)-based nucleases (CRISPR/Cas9) [90, 91]. ZFNs and TALENs work as heterodimers, with each monomer consisting of multiple DNA binding domains and a partial Fok1 nuclease. The DNA binding domains of ZNFs are tandem arrays of C2H2 zinc fingers, with each finger recognizing 3-bp of DNA; ZFNs are created such that each half of the heterodimer recognizes between 9 and 18 bp of DNA at the target cut site. The DNA binding domains of TALENs are composed of a tandem array of repetitive 33–35 amino acid modules, with each module recognizing 1-bp of DNA; TALENs are usually created such that they recognize between 12 and 19 bp of DNA at the target cut site. Binding of a pair of heterodimeric ZFNs or TALENs at target sequences leads to Fok1 dimerization and DNA double strand breaks (DSB) [92]. Because TALENs can be assembled based on a single bp recognition schema, they can be targeted to a larger percentage of the genome than can ZFNs, which are based on a 3-nt motif schema. Also, the DNA binding domains of TALENs are easier to assemble than are zinc finger domains. In contrast to ZFNs and TALENs, which rely on protein-target DNA interaction, CRISPR/Cas9 nucleases use complementary binding between RNA and DNA [91]. The most widely used CRISPR/Cas9 system has two components; the Cas9 nuclease and a guide RNA (sgRNA) that can bind to a specific target DNA sequence and recruit Cas9 to that genomic location, resulting in a DSB [91, 93]. Construction of CRISPRs only requires cloning RNA sequences that will hybridize to target sites [90]. A recent study has shown that both the TALE and CRISPR/Cas9 platforms are remarkably specific in both DNA binding and gene regulation and, importantly, can be successfully targeted to closed chromatin [94]. Because of the ease of cloning, reports of high targeting specificity [90], and accessibility of the guide RNAs to regions of methylated DNA [95], most investigators have begun using the CRISPR/Cas9 system to make DSBs in human cells [91].

Deletion of regulatory elements by ZFNs, TALENs, or CRISPR nucleases requires targeting functional nucleases (heterodimeric in the case of ZFNs or TALENs or monomeric in the case of CRISPR nucleases) to both sides of the element. DSBs occur at both target sites, resulting in local sequence alterations at each target site and loss of the intervening sequences. Recent studies have shown that genomic regions ranging from several bp to more than 1 Mb can be deleted [96–99], with deletion efficiency having an inverse correlation with the size of the deleted region [100]. The frequency of obtaining biallelic deletions in normal cells having diploid chromosome numbers is much higher than when multi-copy genomic regions (created by amplification or increased chromosomal copy numbers) in cancer cells are targeted. In most cases, many clones must be analyzed to identify cells that lack all copies of the regulatory element under study. It is also important to keep in mind that if a regulatory element plays a large role in controlling expression of an essential gene, then deletion of all copies of that element from the genome could affect cell proliferation or survival [100]; in this case, cells having monoallelic or partial loss of the copies of the element (in the case of aneuploid cancer cells) must be analyzed. Several recent studies have used genomic nucleases to delete regulatory elements and identify target genes. For example, Li et al. deleted a 13 Kb section of an enhancer located 100 Kb downstream of the *Sox2* gene and observed ~90 % downregulation of *Sox2* gene expression [98]. Myer et al. deleted a Vitamin D receptor (VDR) binding region located 10 Kb upstream of the *Mmp13* gene and found that VDR-mediated regulation of *Mmp13* was abolished. They also deleted a RUNX2 binding region located 30 Kb upstream of *Mmp13* and observed a complete loss of Mmp13 expression [101]. Hnisz et al. deleted several individual H3K27Ac peaks within a large enhancer in embryonic stem cells and showed that each individual constituent modestly contributed to expression of a target gene [102]. Deletion of enhancers in human colon cancer cells has shown variable effects on the transcriptome. For example, deletion of enhancers that have colon cancer-associated SNPs resulted in the downregulation of hundreds of genes; in contrast, deletion of an enhancer lacking colon cancer-associated SNPs affected very few genes (Y.G. Tak and P.J. Farnham, unpublished data).

An alternative method to identify target genes for distal Candidate Functional SNPs is to modulate the chromatin state of the element using ZFPs or TALEs fused to a chromatin modifying domain or by using a “dead” Cas9 that has no nuclease activity (dCas9) fused to a chromatin modifying domain; such engineered systems are termed “epigenetic toggle switches.” To mimic deletion of an enhancer, epigenetic repressors can be employed. The lysine-specific histone demethylase KDM1A (also known as LSD1) and a KRAB domain that recruits the KAP1/SETDB1 histone methylase have been fused to TALEs and dCAS9; constructs having KDM1A should decrease active histone methylation marks whereas constructs having the KRAB domain should increase inactivating histone methylation marks. One study has suggested that dCas9-KRAB is more efficient than TALE-KRAB for inactivating enhancers, perhaps due to steric hindrance caused by bound dCas9 in preventing recruitment of activating factors [103]. Another study that targeted dCas-LSD1 to the distal enhancer of Oct4 and Tbx3 showed loss of H3K4me2 and a dramatic decrease of H3K27Ac at enhancer regions. Interestingly, the action of dCas9-LSD1 was shown to be specific to enhancers, with very little consequences if targeted to promoters. In contrast, in another study dCas9-KRAB was more effective at promoters, resulting in an increase of H3K27me3 or H3K29me3 level at targeted promoters but not at targeted enhancers [104].

To achieve the opposite effect, investigators have used domains such as VP64, an activating domain that recruits HATs, as well as the enzymatic domain of the p300 HAT to increase the levels of active epigenetic marks at regulatory elements. Gao et al. modified enhancers that regulate the *Oct 4* gene using either TALE-VP64 or dCas9-VP64. These enhancers are normally only active in embryonic stem cells and are marked by the repressive histone modification H3K27me3 in mouse embryonic fibroblasts. They found that dCas9-VP64 less robustly activates the *Oct4* enhancers compared to TALE-VP64; in addition, TALE-VP64 constructs targeted to these enhancers decreased levels of H3K27me3 and increased levels of the active marks H3K27Ac and H3K4me1 [103]. Polstein et al. used TALE-VP64 and dCas9-VP64 for comparison in genome-wide DNA binding, gene expression, and DHS-seq [94]. Although both platforms demonstrated high specificity in DNA binding and gene expression assay, there were several differences. Namely, ChIP-seq signals at the target sites were higher for dCas9-VP64 than for TALE-VP64, whereas gene expression was greater using TALE-VP64. The authors speculate that perhaps the dissociation of genomic DNA caused by the RNA-DNA interactions mediated by the guide RNA affected nearby transcription complexes; they suggest that new dCas9-based activator platforms may show more robust transcriptional activity [105]. A recent study showed that the catalytic domain of the HAT P300 (P300^core^) fused to dCas9 could activate target enhancers and promoters. In this study, a single gRNA targeting an enhancer region with dCas9-P300 ^core^ was sufficient to activate target gene expression, whereas other dCas9 activators required several gRNAs to achieve high levels of gene expression [106]. The authors suggested that the P300 domain may be superior to the VP64 domain because P300 directly regulates histone acetylation whereas VP64 must recruit a HAT. It is possible that many of the differences in effectiveness of the various activating or repressing epigenetic toggle switches in the different studies are due to specific features of the exact promoters and enhancers that were studied. However, considering the ease of cloning guides RNAs, it seems that CRISPR/dCas9 constructs such as dCas9-P300^core^ and dCas9-LSD1 could become a standard method used to identify target genes after turning on repressed enhancers or turning off activated enhancers, respectively.

*Specific targeting of Candidate Functional SNPs* Once deletion or epigenetic modification of a distal regulatory element has been shown to have functional consequences, a more detailed analysis can be performed to compare the effects of the risk and non-risk alleles and to identify specific nucleotides within the element important for regulation; this same approach can be used to study the effect of a SNP on the activity of a promoter region (Fig. 1; Approach B). In traditional approaches, investigators have used luciferase reporter assays to test individual TF binding sites of enhancers. Such studies require removing putative enhancer elements from their native chromosomal structure and location and ligating them into luciferase constructs such that they regulate a heterologous promoter [107, 108]. In addition to not using the correct promoter to test enhancer elements, the choice of cell type could influence the results for enhancers, which function in a highly cell-type specific manner. Another approach using mice involves pronuclear injection of endogenous versus mutated enhancer sequences linked to a lacZ gene [109]. These approaches have issues regarding copy number and position-dependent effects on reporter gene activity and effects of foreign DNA sequences on the native genomic landscape that perturb endogenous gene expression [109]. More recent studies have used genomic engineering to compare endogenous versus mutated regulatory elements. When CRISPR/Cas9 makes a double stranded break, cells use either nonhomologous end-joining (NHEJ) or homology-directed repair (HDR) to repair the break [91]. DNA repair mediated by NHEJ is used when two CRISPR nucleases are targeted to either side of an enhancer, resulting in local alterations at each target site and loss of the intervening sequences. However, because NHEJ results in small insertions or deletions at the site of cleavage this method can also be used for disrupting TF motifs if one guide RNA is precisely targeted to the motif. Another way to study the precise effects of removing or altering a SNP is to substitute a section of the genome with exogenously provided DNA, using the HDR pathway. By providing, along with the guide RNAs and Cas9, a donor DNA fragment that is basically identical to the genomic sequence but contains the alternative SNP allele or a mutation of a TF motif, a precise exchange of genomic regions can be accomplished.

In one study, Vierstra et al. deleted three DHSs located 62, 58, and 55 Kb away from the TSS of the *BCL11A* gene, which encodes a TF that represses fetal hemoglobin (HbF) levels. Deletion of the DHSs located at 55 and 58 Kb away using TALENs led to downregulation of *BCL11A* and increased level of HbF, but no effect was seen after deletion of the DHS located 62 Kb away [110]. This study provides an excellent example that demonstrates the utility of deleting regulatory elements prior to performing more detailed mutational analyses of an element. In this case, studies of individual binding sites in the DHS located 62 Kb away would have not been useful. Following upon the deletion studies, Viestra et al. then used ZFNs to disrupt five TF footprints in the enhancer located 58 Kb away from *BCL11A* and found that disruption of one of the TF footprints led to reduction of *BCL11A*. Another method for identifying critical regions of an enhancer is to use tiled guide RNAs with Cas9. Investigators used ~150 to ~200 different guide RNAs to target the +55, +58, and +62 DHS regions of the *BCL11A* locus. They found that guide RNAs that disrupted the +58 DHS showed the most effect on gene expression [111]. Even though HDR is less efficient compared to NHEJ, the fact that this mechanism can be used to exchange DNA fragments between a plasmid and the genome makes this the method of choice to study SNP-specific differences. Several studies have used CRISPR/Cas9 and HDR-mediated genome editing to change SNPs in mice and cell culture model systems [112–116]. The most common method is to introduce plasmids that express the guide RNAs and Cas9, along with a plasmid that contains the donor sequence (e.g., an enhancer fragment that has the SNP changed to the other allele). Claussnitzer et al. transfected guide RNAs along with Cas9 and donor DNA plasmids into cultured adipose cells to switch a T2D risk SNP to the non-risk SNP allele, affecting binding of a TF and causing a decrease in target gene expression [116]. Other studies have reported an increased efficiency of HDR-mediated genome editing using purified guide RNAs and Cas9 mRNA in place of the expression plasmids and single stranded oligodeoxynucleotides having homology arms in place of the double stranded DNA [117, 118]. Using this strategy in a mouse model, Han et al. substituted a 5-nt sequence within an intronic region of the *Cnn1* gene, which disrupted a CArG box for SRF and caused a reduction in expression of *Cnn1* [112]. Finally, these genomic tools can be used to study orientation dependence of a region harboring a Candidate Functional SNP. CTCF-mediated loops are frequently formed in a convergent orientation involving homodimerization of CTCF proteins located quite far apart on the genome, with the orientation of the CTCF sites determining the choice of interaction between specific enhancers and promoters [119, 120]. Using 2 guide RNAs and Cas9, Guo et al. inverted the region containing a CTCF binding site, switching the CTCF orientation with respect to surrounding CTCF sites; they found that this inversion resulted in changes in gene expression patterns [119].

### Disease-related functional analyses

As described above, an integrated and ordered approach should be used to investigate the role of non-coding SNPs in gene expression. Namely, after SNP prioritization, a combination of deletion or modification of a regulatory element plus eQTL analyses can provide a list of candidate target genes. However, an analysis of non-coding risk-associated SNPs is not complete without further characterization of how genes whose activity is influenced by a particular SNP affect initiation, progression, or manifestation of the disease under study. Identifying the causal gene(s) will provide insights into the disease and perhaps also provide new diagnostic or therapeutic targets.

It is likely that manipulation of a regulatory element or eQTL analyses will identify more than one candidate target gene. Thus, it may be difficult to know which of the genes whose expression is linked to the SNP should be tested in phenotypic assays. Investigators often choose putative causal genes based on (a) proximity to the regulatory element, (b) degree to which expression is affected, or (c) a gene function that can be easily imagined to contribute to the disease risk. Each of these choices is fraught with problems. For example, as discussed above, genes are not necessarily near their regulatory elements. Another confounding issue is that changes in mRNA do not always lead to similar changes in protein levels [121, 122] and thus the genes that show the largest changes in mRNA might not necessarily produce the largest changes in protein. Finally, gene function is often assigned based on the first set of experiments performed on that gene; many genes function in multiple networks, often in a tissue-specific manner. Therefore, it is important to keep in mind that identifying a causal gene may require testing several different candidates. If one of the candidate target genes is tested with negative results, this could mean either that the candidate SNP is not really linked to the disease, that the wrong assay was used, or that the wrong candidate gene was assayed. One approach to deal with this uncertainty is to first develop a functional assay in which effects can be observed upon deletion or modification of the SNP-harboring element; if the element can be shown to affect a particular cellular phenotype, then individual candidate target genes can subsequently be studied using that same assay. For example, Claussnitzer et al. examined the effects of CRISPR-Cas9-mediated editing on cellular signatures of obesity. By changing the risk allele to the non-risk allele, they observed an increase in the basal metabolic rate and increased thermogenesis, supporting the concept that manipulation of a regulatory element can provide important physiological information without knowing the exact target gene [116].

If studying GWAS loci related to cancer, methods that are used for functional follow-up studies include proliferation and cell migration assays [18]. However, cultured cancer cell lines are not ideal model systems because of their genomic instability (which leads to variable karyotypes) and because isolated cancer cell lines grown in tissue culture dishes do not properly represent the complex environment of the cells in the context of either a normal tissue or a tumor. Investigators have begun to use 3 dimensional organoids [123], normal cell lines, or isogenic ES or iPS cells [124, 125] to try to reproduce a more natural cellular environment for functional studies. However, even these assays do not allow the study of effects seen only within a complex tissue. If a mouse model exists that closely reproduces the human disease, then perhaps this would be the ideal system to use; the phenotypic influence of a SNP and/or putative causal target gene may be more consequential in a living organism than in a short-term cell culture assay. For example, when a mouse lacking a homologous enhancer that is associated with colon cancer in humans was crossed to a mouse that spontaneously develops tumors in the intestine and colon, the incidence of polyp formation was reduced in their offspring [88]. Another issue to consider is that an individual SNP or regulatory element may not cause dramatic phenotypic differences. Instead, it may be necessary to study combinations of SNPs. A recent report evaluating the combinatorial effects of SNPs showed that different SNPs in the same LD block identified different enhancers that cooperatively regulate the same target gene [126]. Such studies suggest that altering an individual GWAS-identified regulatory element may have fewer functional consequences than inactivation of a target gene. However, if multiple target genes work together to contribute to disease risk then even moving from SNP to target gene may not solve the problem. Perhaps investigators could use multiplexing CRISPR/Cas9 systems [127–129] to simultaneously target many regulatory elements and/or putative target genes from several different risk-associated loci to test for combinatorial effects in phenotypic assays [130].

If an appropriate assay is identified whose outcome is influenced by loss or modification of the SNP or regulatory element, then candidate target genes can be tested using that same assay in the hopes of identifying the causal gene. Commonly used approaches to investigate the function of a candidate causal gene include overexpressing an exogenous form of the gene (e.g., using a cloned cDNA) or reducing levels of the endogenous gene using RNAi tools [131]. In a recent study of the FTO locus, which is related to T2D, Claussnitzer et al. identified SNPs in an enhancer that is only active during early adipocyte differentiation and showed that the expression of candidate target genes (IRX3 and IRX5) correlated with the presence of the risk-allele haplotype. Cells harboring the risk allele showed increased thermogenesis, a hallmark of obesity. The investigators showed, using primary preadipocytes isolated from risk-allele carriers, that reducing levels of IRX3 or IRX5 restored thermogenesis to non-risk levels and that overexpression of IRX3 in preadipocytes that contain non-risk allele produced the opposite effect [116]. Another group used an IRX3 knockout mouse as well as mice conditionally expressing a dominant negative form of IRX3 to demonstrate a link between the relationship of IRX3 to body mass and energy homeostasis [132]. More recently, alternative approaches for overexpressing or repressing genes have been developed that are based on the genomic engineering tools described above. For example, investigators have used CRISPR/Cas9 nucleases to mutate coding regions [133] and epigenomic tools such as TALEs and dCAs9 fused to activator or repression domains have been used to regulate the promoter of a gene of interest [134]. However, it is important to consider that overexpressing a gene from a cDNA may not appropriately provide the correct splice variant [135] and that inactivation methods such as siRNA, shRNA, or genomic nucleases have the inherent problem of off-target effects [136].

## Conclusions

As detailed within, investigators are making great strides toward understanding the functional relevance of non-coding SNPs and how they can contribute to disease risk. With the advent of new genome engineering tools, putative target genes are now being associated with GWAS index SNPs for a variety of diseases. However, a limitation of the genomic and epigenomic editing technologies described above is that it is hard to distinguish target genes directly regulated by a risk-associated enhancer from genes whose expression has been indirectly affected as a consequence of the expression changes of the direct targets. For example, changes in expression of a TF can lead to subsequent changes in expression of genes regulated by that TF and changes in expression of a kinase can lead to alterations of many components of critical signaling pathways. A recent study has shown that deletion of a single enhancer in colon cancer cells can lead to changes in expression of hundreds of genes, most likely due to the fact that the direct target gene regulated by that enhancer is the *MYC* oncogene (Tak and Farnham, unpublished data).

One approach that can be used to distinguish genes that are directly vs. indirectly affected by a risk-associated enhancer is to perform physical interaction assays. Many interaction assays are based on principles of the chromosome confirmation capture (3C) assay, which involves capturing chromosome interactions by formaldehyde cross-linking, followed by digestion with a restriction enzyme and subsequent ligation of DNA regions that were brought together by protein–protein interactions; ligation frequency between two loci is assessed using qPCR [137]. Using 3C, Zhang et al. investigated all possible interactions between a prostate-specific enhancer and genes that are within an ~3 Mb window, identifying a single loop to a gene that is 1 Mb away from the enhancer [138]. However, the results from 3C assays are limited to a pre-selected region, excluding the discovery of interactions with regions beyond the tested genomic window. A modification of 3C, circular chromosome conformation capture followed by sequencing (4C-seq), allows the investigation of all possible interactions mediated by a specific enhancer by employing high-throughput sequencing instead of qPCR. Using 4C-seq, investigators showed that enhancers located within an intron of the *FTO* gene and harboring obesity and T2D GWAS-identified SNPs do not interact with the *FTO* promoter but instead interact with the *IRX3* gene which is located 500 Kb downstream [132]. Hi-C, another variation of 3C, can be used to study all chromatin interaction within the genome. Unfortunately, the majority of Hi-C experiments capture interactions separated by at least 1 Mb [130] and thus may miss nearby enhancer-promoter loops. However, a recent modification of Hi-C, called Capture Hi-C, which increases the resolution of the mapped interactions, has been used to study colon cancer risk SNPs. These experiments identified interactions that are enriched with colon cancer-specific TF binding sites [139]. This technique was also used to identify short-range interactions between an enhancer and a gene 26 Kb away [140]. Therefore, to study interactions between enhancers and promoters, investigators should consider methods such as Capture-C [139] or HiCap [86] since they not only provide better resolution, but also can detect hundreds of interactions in one experiment. Importantly, even though looping assays that identify interactions between regulatory elements harboring SNPs and promoters can provide clues as to the identity of putative target genes, it is important to compare these results to those in which the regulatory element has been experimentally deleted or modified. Genes whose expression levels are linked to the regulatory element and that are also involved in promoter-enhancer loops are likely to be direct targets, whereas genes whose expression levels are linked to the element but no loops are found can either be indirect targets or direct targets that are difficult to identify due to limitations of the current looping assays; it is also possible that enhancer-promoter loops will be identified that are not related to genes whose expression changes upon manipulation of the enhancer.

Finally, it is important to return to the overarching reason as to why GWAS experiments are performed, i.e., a desire to have a better understanding of the set of genes that contribute to increased risk for a particular disease. It is important to keep in mind that a gene whose expression is indirectly affected by a non-coding SNP could be a more important diagnostic or therapeutic target that the direct target gene. Thus, it is critical to identify both the direct targets of a risk-associated regulatory element and other genes affected by reduction of levels of the direct targets. This requires genomic manipulation with subsequent gene expression analyses; looping assays cannot identify indirect targets or affected signaling pathways. Identifying a therapeutic agent against either a direct or an indirect target gene that could dampen the phenotypic consequences (i.e., increased disease risk) conferred by the risk-associated SNP would provide a wonderful molecular solution to studies that begin with epidemiological population analyses.

## Abbreviations

ATAC-Seq: assay of transposase accessible chromatin high-throughput sequencing
CAGE: cap analysis of gene expression
CRISPR: clustered regularly interspaced short palindromic repeat
CTCF: CCCTC-binding factor
DHS: DNase1 hypersensitive region
DSB: DNA double strand break
ENCODE: Encyclopedia of DNA Elements
eRNA: enhancer RNA
eQTL: expression quantitative trait loci
GWAS: Genome Wide Association Studies
HAT: histone acetylases
HbF: fetal hemoglobin
HDR: homology-directed repair
H3K4me1: histone H3 monomethylated on lysine 4
H3K27Ac: histone H3 acetylated on lysine 27
H3K4me3: histone H3 trimethylated on lysine 4
FANTOM5: functional annotation of the mammalian genome 5
LD: linkage disequilibrium
LDL-C: low-density lipoprotein cholesterol
LSD1: lysine-specific histone demethylase KDM1A
meQTL: methylation QTL
MI: myocardial infarction
NHEJ: nonhomologous end-joining
Reg SNPs: regulatory SNPs
REMC: roadmap epigenomics mapping consortium
SNP: single-nucleotide polymorphism
sgRNA: guide RNA
TAD: topologically associating domain
TALE: transcription activator-like effector
TALEN: TALE-based nuclease
TF: transcription factor
TSS: transcription start site
T1D: type 1 Diabetes
T2D: type 2 Diabetes
VDR: vitamin D receptor
VP64: the quadruple tandem repeat of the herpes simplex virus VP16
ZFN: zinc finger-based nuclease
ZFP: zinc finger protein
3C: chromosome confirmation capture
4C-seq: circular chromosome conformation capture followed by sequencing
